# Study protocol of the Asian XELIRI ProjecT (AXEPT): a multinational, randomized, non-inferiority, phase III trial of second-line chemotherapy for metastatic colorectal cancer, comparing the efficacy and safety of XELIRI with or without bevacizumab versus FOLFIRI with or without bevacizumab

**DOI:** 10.1186/s40880-016-0166-3

**Published:** 2016-12-22

**Authors:** Masahito Kotaka, Ruihua Xu, Kei Muro, Young Suk Park, Satoshi Morita, Satoru Iwasa, Hiroyuki Uetake, Tomohiro Nishina, Hiroaki Nozawa, Hiroshi Matsumoto, Kentaro Yamazaki, Sae-Won Han, Wei Wang, Joong Bae Ahn, Yanhong Deng, Sang-Hee Cho, Yi Ba, Keun-Wook Lee, Tao Zhang, Taroh Satoh, Marc E. Buyse, Baek-Yeol Ryoo, Lin Shen, Junichi Sakamoto, Tae Won Kim

**Affiliations:** 1 Gastrointestinal Cancer Center, Sano Hospital, Hyogo, 655-0031 Japan; 2 State Key Laboratory of Oncology in South China, Department of Medical Oncology, Collaborative Innovation Center for Cancer Medicine, Sun Yat-sen University Cancer Center, Guangzhou, Guangdong 510060 P. R. China; 3 Department of Clinical Oncology, Aichi Cancer Center Hospital, Nagoya, 464-8681 Japan; 4 Department of Medicine, Samsung Medical Center, Sungkyunkwan University School of Medicine, Seoul, 135-710 South Korea; 5 Department of Biomedical Statistics and Bioinformatics, Graduate School of Medicine, Kyoto University, Kyoto, 606-8501 Japan; 6 Gastrointestinal Medical Oncology Division, National Cancer Center Hospital, Tokyo, 104-0045 Japan; 7 Department of Surgical Specialties, Graduate School, Tokyo Medical and Dental University, Tokyo, 113-8519 Japan; 8 Department of Gastrointestinal Medical Oncology, National Hospital Organization Shikoku Cancer Center, Matsuyama, 791-0280 Japan; 9 Department of Surgical Oncology, The University of Tokyo, Tokyo, 113-0033 Japan; 10 Department of Surgery, Tokyo Metropolitan Komagome Hospital, Tokyo, 113-8677 Japan; 11 Division of Gastrointestinal Oncology, Shizuoka Cancer Center, Shizuoka, 411-8777 Japan; 12 Department of Internal Medicine, Seoul National University Hospital, Seoul, 110-744 South Korea; 13 Department of Gastrointestinal Oncology, The First People’s Hospital of Foshan, Foshan, Guangdong 528000 P. R. China; 14 Department of Internal Medicine, Yonsei University College of Medicine, Seoul, 120-752 South Korea; 15 Department of Medical Oncology, The Sixth Affiliated Hospital, Sun Yat-sen University, Guangzhou, Guangdong 510655 P. R. China; 16 Department of Hematology-Oncology, Chonnam National University Medical School, Gwangju, 519-809 South Korea; 17 Department of Digestive Oncology, Tianjin Medical University Cancer Institute and Hospital, Tianjin, 300060 P. R. China; 18 Department of Internal Medicine, Seoul National University Bundang HospitalSeoul National University College of Medicine, Seongnam, 463-707 South Korea; 19 Department of Medical Oncology, Union Hospital of Tongji Medical College, Huazhong University of Science and Technology, Wuhan, Hubei 430022 P. R. China; 20 Department of Gastroenterological Surgery, Graduate School of Medicine, Osaka University, Osaka, 565-0871 Japan; 21 International Drug Development Institute, Louvain-La-Neuve, 1340 Belgium; 22 Department of Oncology, Asan Medical Center, University of Ulsan Collage of Medicine, Seoul, 138-736 South Korea; 23 Department of Gastrointestinal Oncology, Peking University Cancer Hospital & Institute, Beijing, 100-142 P. R. China; 24 Tokai Central Hospital, Kakamigahara, 504-8601 Japan

**Keywords:** Metastatic colorectal cancer, Randomized phase III clinical trial, XELIRI, Bevacizumab, Second-line therapy

## Abstract

**Background:**

Capecitabine and irinotecan combination therapy (XELIRI) has been examined at various dose levels to treat metastatic colorectal cancer (mCRC). Recently, in the Association of Medical Oncology of the German Cancer Society (AIO) 0604 trial, tri-weekly XELIRI plus bevacizumab, with reduced doses of irinotecan (200 mg/m^2^ on day 1) and capecitabine (1600 mg/m^2^ on days 1–14), repeated every 3 weeks, has shown favorable tolerability and efficacy which were comparable to those of capecitabine and oxaliplatin (XELOX) plus bevacizumab. The doses of capecitabine and irinotecan in the AIO trial are considered optimal. In a phase I/II study, XELIRI plus bevacizumab (BIX) as second-line chemotherapy was well tolerated and had promising efficacy in Japanese patients.

**Methods:**

The Asian XELIRI ProjecT (AXEPT) is an East Asian collaborative, open-labelled, randomized, phase III clinical trial which was designed to demonstrate the non-inferiority of XELIRI with or without bevacizumab versus standard FOLFIRI (5-fluorouracil, leucovorin, and irinotecan combination) with or without bevacizumab as second-line chemotherapy for patients with mCRC. Patients with 20 years of age or older, histologically confirmed mCRC, Eastern Cooperative Oncology Group performance status 0–2, adequate organ function, and disease progression or intolerance of the first-line regimen will be eligible. Patients will be randomized (1:1) to receive standard FOLFIRI with or without bevacizumab (5 mg/kg on day 1), repeated every 2 weeks (FOLIRI arm) or XELIRI with or without bevacizumab (7.5 mg/kg on day 1), repeated every 3 weeks (XELIRI arm). A total of 464 events were estimated as necessary to show non-inferiority with a power of 80% at a one-sided α of 0.025, requiring a target sample size of 600 patients. The 95% confidence interval (CI) upper limit of the hazard ratio was pre-specified as less than 1.3.

**Conclusion:**

The Asian XELIRI ProjecT is a multinational phase III trial being conducted to provide evidence for XELIRI with or without bevacizumab as a second-line treatment option of mCRC.

*Trial registration* ClinicalTrials.gov NCT01996306. UMIN000012263

## Background

Life-prolonging systemic therapies, e.g., chemotherapies with or without molecular targeted agents such as anti-vascular endothelial growth factor (VEGF) or anti-epidermal growth factor receptor (EGFR) agents, are important for unresectable metastatic colorectal cancer (mCRC). The National Comprehensive Cancer Network (NCCN) guidelines [1], the European Society for Medical Oncology (ESMO) clinical practice guidelines [2], and the Japanese Society for Cancer of the Colon and Rectum (JSCCR) guidelines [3] recommend four basic cytotoxic chemotherapy regimens as options to patients with mCRC who are able to tolerate intensive therapy.

Recently, head-to-head randomized phase III studies comparing bevacizumab and cetuximab (e.g., FIRE-3 and CALGB80405) did not show a consistent substantial difference in response rate, overall survival (OS), or progression-free survival (PFS) [4–6]. A randomized phase III study (STRATEGIC-1) that was designed to determine the best sequence of systemic therapy is now in progress [7].

Combination chemotherapy using oral drugs is convenient, freeing patients from chemoports or infusion pumps. However, compelling evidence for the safety and efficacy of such regimens is required.

In a phase III BICC-C study conducted mainly in the United States, tri-weekly XELIRI regimen (also named CapeIRI regimen: intravenous infusion of irinotecan 250 mg/m^2^ on day 1 and oral administration of capecitabine 2000 mg/m^2^ per day on days 1–15) was compared with FOLFIRI regimen (intravenous infusion of irinotecan 180 mg/m^2^, leucovorin [LV] 400 mg/m^2^, and 5-fluorouracil [5-FU] 400 mg/m^2^ on day 1 followed by a 46-hour infusion of 5-FU 2400 mg/m^2^, repeated every 2 weeks) and modified IFL regimen (intravenous infusion of irinotecan 125 mg/m^2^ on day 1, LV 20 mg/m^2^ and 5-FU 500 mg/m^2^ on days 1 and 8, repeated every 3 weeks) [8]. Grade 3/4 adverse events mainly consisting of gastrointestinal toxicities occurred more frequently in patients treated with CapeIRI than in those treated with FOLFIRI (nausea, 18.4% vs. 8.8%; diarrhea, 47.5% vs. 13.9%; dehydration, 19.1% vs. 5.8%); median PFS was significantly shorter for patients treated with CapeIRI than for those treated with FOLFIRI (5.8 vs. 7.6 months, *P* = 0.015) due to early discontinuation of CapeIRI regimen. The authors suggested that the large number of patients with early treatment discontinuations for adverse events in the CapeIRI group may because of regional and ethnic differences in the metabolism of 5-FU and capecitabine, especially between patients in the United States and East Asia [8, 9]. Subsequently, a modified XELIRI regimen, with reduced doses of irinotecan and capecitabine, was studied in combination with bevacizumab, mainly in studies comparing FOLFIRI and XELOX regimens (intravenous infusion of oxaliplatin 130 mg/m^2^ on day 1 plus oral administration of capecitabine 2000 mg/m^2^ per day on days 1–15) [10–13].

Recently, in the AIO 0604 trial, tri-weekly XELIRI plus bevacizumab, with reduced doses of irinotecan (200 mg/m^2^ on day 1) and capecitabine (1600 mg/m^2^ per day on days 1–14), was compared with XELOX plus bevacizumab [13]. Common grade 3/4 adverse events included diarrhea (16% for the XELIRI arm and 22% for the XELOX arm), nausea (3% for each arm), and fatigue (3% for each arm). The median PFS was 12.1 vs. 10.4 months [13]. This randomized phase II trial showed that XELIRI plus bevacizumab had equivalent efficacy to XELOX plus bevacizumab, even though the XELIRI-based regimen was designed primarily to reduce adverse events.

In Japan, a completed phase I/II study evaluated the efficacy of the XELIRI regimen using the same dose in the AIO 0604 trial for patients with mCRC who had previously been treated with oxaliplatin and bevacizumab (the BIX study) [14]. The most common grade 3/4 adverse events were neutropenia (8.8%), nausea (5.9%), diarrhea (5.9%), and fatigue (2.9%). The efficacy analysis demonstrated an overall response rate of 17.6% and median PFS of 8.3 months. These results suggest that XELIRI plus bevacizumab would be safe and effective for East Asian patients with mCRC. Considering that there are regional differences between the United States and East Asian patients with respect to the metabolism of capecitabine and 5-FU and that gastrointestinal toxicities may be more tolerable for East Asian patients, XELIRI plus bevacizumab may be more appropriate for Asian patients [9]. Therefore, XELIRI with or without bevacizumab was assigned to the study therapy group in the AXEPT trial.

Homozygosity or double heterozygosity for UDP-glucuronosyl transferase 1A1 ( *UGT1A1* ) polymorphisms (UGT1A1*28 and UGT1A1*6) may relate to increased serious adverse events, such as neutropenia and diarrhea, in patients treated with an irinotecan-based regimen. A reduced dose of irinotecan is therefore needed in patients with these polymorphisms [15–19].

## Methods

### Objectives

The primary objective is to demonstrate the non-inferiority in terms of OS for XELIRI with or without bevacizumab versus FOLFIRI with or without bevacizumab as second-line therapy for patients with mCRC.

The secondary objectives are to evaluate the PFS, time to treatment failure (TTF), overall response rate (ORR), disease control rate (DCR), relative dose intensity (RDI), safety, and association between *UGT1A1* polymorphisms and the occurrence rates of adverse events. Exploratory subgroup analysis is planned to investigate factors which are thought to influence prognosis, including country, *Kirsten rat sarcoma viral oncogene homolog (KRAS)* genotype, and *UGT1A1* genotype.

XELIRI improves treatment convenience by eliminating continuous intravenous infusion and permitting fewer hospital visits (every 3 weeks). Therefore, demonstration of the non-inferiority of XELIRI with or without bevacizumab versus FOLFIRI with or without bevacizumab will provide new evidence for this novel treatment option as second-line therapy for mCRC.

### Trial design

AXEPT is an East Asian collaborative, open-labelled, randomized, non-inferiority, phase III clinical trial comparing the efficacy and safety of XELIRI with or without bevacizumab versus FOLFIRI with or without bevacizumab in patients with mCRC.

After written informed consent has been obtained, eligible patients will be randomized (1:1) to either the XELIRI arm or the FOLFIRI arm using minimization methods by the Swedish central electronic case-report form system (eCRF: VIEDOC®, PCG Solutions Co. Ltd., Uppsala, Sweden). Stratification factors will include (1) country (Japan vs. South Korea vs. China), (2) Eastern Cooperative Oncology Group performance status (ECOG PS) (0–1 vs. 2), (3) number of metastatic sites (1 vs. >1), (4) prior oxaliplatin treatment (yes vs. no), and (5) concomitant bevacizumab treatment (with vs. without).

**Fig. 1:**
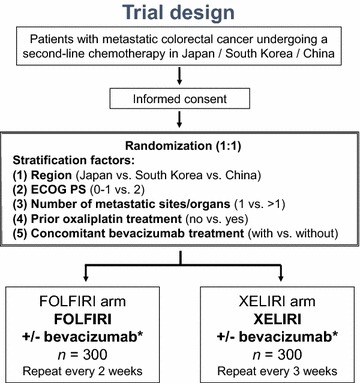
Study design of the Asian XELIRI ProjecT (AXEPT). *All patients from South Korea and Japan will receive concomitant bevacizumab treatment; whereas those from China will be treated either with or without concomitant bevacizumab treatment. *ECOG PS* Eastern Cooperative Oncology Group performance status. The FOLFIRI arm: intravenous infusion of irinotecan 180 mg/m^2^, leucovorin 400 mg/m^2^, and 5-fluorouracil 400 mg/m^2^ on day 1 followed by a 46-h infusion of 5-fluorouracil 2400 mg/m^2^, repeated every 2 weeks. The XELIRI arm: intravenous infusion of irinotecan 200 mg/m^2^ on day 1 and oral administration of capecitabine 1600 mg/m^2^ per day on days 1–14, repeated every 3 weeks

The study will be conducted in three countries, Japan, South Korea, and China (Fig. 1). The steering committee consists of a principal investigator from each country and a biostatistician. A total of 600 patients will be enrolled from 73 Japanese hospitals, 8 South Korean hospitals, and 17 Chinese hospitals.

### Study population

Patients who meet all of the below inclusion criteria and none of the exclusion criteria will be eligible for enrollment in this study.

#### Inclusion criteria

1. Histologically confirmed unresectable colorectal adenocarcinoma
2. Age ≥20 years
3. ECOG PS 0–2
4. Signed and dated written informed consent
5. Life expectancy >90 days
6. Withdrawal from first-line chemotherapy (regardless of the combination with or without molecular targeted agents) for mCRC due to intolerable toxicity or disease progression, or relapse within 180 days after the last dose of adjuvant chemotherapy
7. Adequate organ functions according to the following laboratory values which are obtained within 14 days before enrollment
  Neutrophil count ≥1500/mm^3^
  Platelet count ≥100 × 10^3^/mm^3^
  Hemoglobin ≥9.0 g/dL
  Total bilirubin ≤1.5 mg/dL
  Aspartate aminotransferase (AST) ≤100 IU/L (100 U/L) (≤200 IU/L [200 U/L] if liver metastases are present)
  Alanine transaminase (ALT) ≤100 IU/L (100 U/L) (≤ 200 IU/L [200 U/L] if liver metastases are present)
  Serum creatinine ≤1.5 mg/dL

#### Exclusion criteria

1. History of other malignancies with a disease-free interval <5 years
2. Massive pleural effusion or ascites requiring intervention
3. Radiological evidence of brain tumor or brain metastases
4. Active infection, including hepatitis
5. Any of the following concurrent diseases:
  Gastrointestinal bleeding or gastrointestinal obstruction (including paralytic ileus)
  Symptomatic heart disease (including unstable angina, myocardial infarction, and heart failure)
  Interstitial pneumonia or pulmonary fibrosis
  Uncontrolled diabetes mellitus
  Uncontrolled diarrhea (that interferes with daily activities despite adequate therapy)
6. Any of the following in the medical history:
  Myocardial infarction (history of one episode within 1 year before enrollment or two or more lifetime episodes)
  Serious hypersensitivity to any of the study drugs
  History of adverse reaction to fluoropyrimidines suggesting dihydropyrimidine dehydrogenase deficiency
7. Previous treatment with irinotecan
8. Current treatment with atazanavir sulfate
9. Previous treatment with tegafur, gimeracil, and oteracil potassium within 7 days before enrollment
10. Pregnant or lactating women, and men or women unwilling to use contraception
11. Requirement for continuous treatment with steroids
12. Psychiatric disability that would preclude study compliance
13. Otherwise determined by the investigator to be unsuitable for participation in the study

Additional exclusion criteria for those receiving bevacizumab:

1. Concurrent gastrointestinal perforation or history of gastrointestinal perforation within 1 year before enrollment
2. History of pulmonary hemorrhage/hemoptysis ≥grade 2 (defined as at least 2.5 mL of bright red blood) within 1 month before enrollment
3. History of laparotomy, thoracotomy, or intestinal resection within 28 days before enrollment
4. Incomplete wound healing (except suture wounds from implantation of a central venous port), gastrointestinal ulcer, or traumatic fracture
5. Current or recent (within 1 year) thromboembolism or cerebrovascular disease
6. Currently receiving or requiring anticoagulation therapy (>325 mg/day of aspirin)
7. Bleeding diathesis, coagulopathy, or coagulation factor abnormality (international normalized ratio [INR] ≥1.5 within 14 days before enrollment)
8. Uncontrolled hypertension
9. Urine protein by dipstick > +2

### Study treatment

Patients will be randomly assigned to receive one of the following treatments:

The FOLFIRI arm: intravenous infusion of irinotecan 180 mg/m^2^, *l*-LV 200 mg/m^2^ (or *d,l*-LV 400 mg/m^2^), bevacizumab 5 mg/kg, and 5-FU 400 mg/m^2^ on day 1 followed by a 46-hour continuous infusion of 5-FU 2400 mg/m^2^, repeated every 2 weeks until disease progression, unacceptable toxicity, or patient withdrawal.

The XELIRI arm: intravenous infusion of irinotecan 200 mg/m^2^ and bevacizumab 7.5 mg/kg on day 1, and oral administration of capecitabine 1600 mg/m^2^ on days 1–14, repeated every 3 weeks until disease progression, unacceptable toxicity, or patient withdrawal.

In both arms, the dose for irinotecan will be started at 150 mg/m^2^ in patients identified to be homozygous for UGT1A1*6 or UGT1A1*28 or double heterozygous for UGT1A1*6 and UGT1A1*28 at baseline screening [20].

All adverse events will be assessed according to the National Cancer Institute Common Toxicity Criteria Adverse Event v4.0 (NCI CTCAE v4.0) [21].

In both arms, the protocol treatment will be started upon the investigator’s decision based on lab values within the inclusion criteria at the start of a treatment cycle. The next cycle should not be administered unless the neutrophil count ≥ 1500/mm^3^, platelet count ≥ 75,000/mm^3^, serum total bilirubin ≤ 1.5 mg/dL, serum creatinine ≤ 1.5 mg/dL, hand-foot syndrome grade ≤ 1, and other non-hematologic toxicities resolve to grade ≤ 1. In those receiving bevacizumab, treatment should not be administered unless hypertension grade ≤ 2, proteinuria ≤ 2+, venous thromboembolism grade ≤ 2, and hemorrhage grade ≤ 1. If the next cycle cannot be started within 28 days of the scheduled start date, the protocol treatment will be discontinued. If adverse events that require dose reduction occur prior to a cycle, dose reduction could be done twice as appropriate. If more than two dose reductions are required, treatment with that drug will be discontinued. Once a dose reduction has been done, the dose should not be increased in subsequent cycles.

### Statistical considerations

The analysis set is the all-randomized population. In the primary analysis, the cumulative OS curve, median OS, and annual OS rate will be estimated with the Kaplan–Meier method, and the confidence interval (CI) of the median OS will be calculated with the Brookmeyer and Crowley method. The point estimates of the hazard ratio (HRs) and their 95% CIs will be calculated with a Cox proportional hazard model.

Secondary endpoints will be analyzed to supplement the results of the primary analysis. No adjustments will be made for multiplicity because the analysis of secondary endpoints is exploratory. Intergroup comparisons will be performed as necessary, but *P* values obtained from statistical tests will be considered reference data only. Exploratory subgroup analysis for factors thought to influence prognosis, including country, *KRAS* genotype, and *UGT1A1* genotype, will be performed.

The sample size of this study was calculated on the basis of two previously reported phase III studies which included FOLFIRI plus bevacizumab or XELIRI plus bevacizumab as second-line treatment of patients with mCRC. In the FIRIS study, the median survival time (MST) was 18.2 months in the FOLFIRI group (13.7 months in patients previously treated with oxaliplatin-based therapy) [22]. In the ML18147 study, the MST was 10.0 months in the group receiving irinotecan-based chemotherapy, such as FOLFIRI and XELIRI, and 12.0 months in the group receiving irinotecan-based chemotherapy plus bevacizumab [23]. The add-on effect of bevacizumab has also been confirmed by phase II studies on Japanese [24], South Korean [25, 26], and Chinese patients [27, 28] and by a retrospective analysis report [29]. In addition, the results of the ML18147 trial [23] and E3200 trial [30] indicate that the add-on effect of bevacizumab in second-line treatment is similar between patients with and without previous treatment of concomitant bevacizumab. Based on the above considerations, we assumed to observe an MST of 11.0 months for patients treated with FOLFIRI and 13.0 months for patients treated with FOLFIRI plus bevacizumab. Due to differences in regional medical environment, all South Korean and Japanese patients will receive concomitant bevacizumab treatment, whereas patients enrolled from China are expected to include those who do not receive concomitant bevacizumab treatment. On the basis of the estimated proportion of patients receiving treatment with or without bevacizumab and the estimated MST for each of these groups, the MST of patients in the FOLFIRI arm was assumed to be 12.6 months. Calculation of the required sample size under these conditions with the following assumptions revealed that an estimated 464 events would be needed to achieve at least 80% power.

(i) HR of treatment to control: 1.00 (MST, 12.6 months for the FOLFIRI arm vs. 12.6 months for the XELIRI arm)
(ii) Upper margin of the non-inferiority hypothesis: HR of 1.30 (MST, 12.6 months for the FOLFIRI arm vs. 9.7 months for the XELIRI arm)
(iii) One-sided significance level: 0.025
(iv) Enrollment period: 24 months
(v) Follow-up period: 18 months

An enrollment of 600 patients, including a 5% annual dropout rate, was therefore set in the study.

The non-inferiority upper margin of HR was set at 1.30 (9.7 months as converted to MST) considering the variation of the 95% CI of MST with stratification by *KRAS* status or therapy with anti-EGFR antibody drugs after the protocol treatment. If the above non-inferiority hypothesis was achieved, the hypothesis will be tested using a non-inferiority upper margin of HR of 1.25.

For sensitivity analysis, Cox regression analysis will also be performed, adding the *KRAS* status as a covariate to avoid the influence of anti-EGFR antibody treatment after the protocol treatment. If necessary, Cox regression analysis will be performed with adjusted demographic factors (for imbalance between the two treatment arms) rather than stratification factors.

### Study coordination and ethical aspects

The study will be conducted according to the protocol in compliance with the principles of the Declaration of Helsinki, International Conference of Harmonization Good Clinical Practice Efficacy 6 (ICH-GCP E6) [31], and the rules and regulations of each country.

The Epidemiological and Clinical Research Information Network (ECRIN) is responsible for study management (including enrollment) and monitoring of Japanese sites and will also assist with and oversee local study management in each data center. ECRIN delegates its responsibility to the South Korean and Chinese local data centers for study preparation, contract, patient enrollment within the designated study term, exchange of safety data, document archives, quality checks/quality assurance, and other local procedures which are stated in the ICH-GCP and local regulations.

The protocol and the informed consent form used in the study must be approved by the Institutional Review Board (IRB)/Independent Ethics Committee (IEC) at each study site prior to the start of study. If IRB/IEC approval is obtained, the site principal investigator will send the copy of IRB/IEC approval document to each data center. The original IRB/IEC approval document will be retained by the site principal investigator, and a copy will be retained at the local data center.

The study protocol was approved by the ECRIN central IRB on September 3, 2013 and was registered at Clinicaltrials.gov (NCT01996306 on November 22, 2013) and UMIN-CTR (UMIN000012263 on November 11, 2013).

In this study, an Independent Data Monitoring Committee (IDMC) is established to determine whether this study is conducted appropriately. The role of the IDMC is to assess at intervals the progress of a clinical trial, the safety data, and the critical efficacy endpoints and to recommend the Steering Committee and study sponsor whether to continue, modify, or stop the trial. IDMC members will not be directly involved in the conduction or operation of the trial.

## Discussion

The XELIRI regimen has already been examined at various doses and combinations since the beginning of the twenty first century [8, 10–14, 25]. The XELIRI regimen used in the AIO 0604 trial is regarded appropriate in terms of efficacy and safety [13]. In addition, according to the ML18147 study [23], 12% of all enrolled patients were treated with the tri-weekly XELIRI plus bevacizumab regimen (AIO XELIRI regimen), and approximately 35% with the irinotecan-based regimen.

However, XELIRI has not been recommended by guidelines (neither from ESMO nor NCCN) because of its toxicities. For that reason, a phase II study (the BIX trial) was conducted to determine the tolerability of the AIO XELIRI regimen in Japanese patients. The results showed that the safety profile was acceptable and the efficacy was promising [14]. Thus, we planned this randomized phase III trial, collaborating with investigators from China and South Korea.

In addition, with regard to *UGT1A1* polymorphisms, it is necessary to evaluate the association between *UGT1A1* polymorphisms and safety or efficacy in East Asian population and to establish clear rules of dose reduction for irinotecan. Thus, we will check *UGT1A1* genotype at the baseline screening and set an initial irinotecan dose for patient with *UGT1A1* polymorphism. The association between *UGT1A1* genotype and safety will be further explored in subgroup analysis.

Demonstration of the non-inferiority of XELIRI with or without bevacizumab to FOLFIRI with or without bevacizumab in our study will provide evidence for a new treatment option as second-line therapy for mCRC.
